# Height, Nutritional and Economic Inequality in Central Spain, 1837–1936

**DOI:** 10.3390/ijerph19063397

**Published:** 2022-03-14

**Authors:** Hector Garcia-Montero

**Affiliations:** Department of Economics and INARBE, Universidad Pública de Navarra, Edificio Los Madroños, Campus de Arrosadia, 31006 Pamplona, Spain; hector.garcia@unavarra.es

**Keywords:** inequality, Spain, height, anthropometrics, nutrition, biological wellbeing, living standards

## Abstract

This article analyzes the evolution of inequality in mean male height in central Spain considering the generations born from 1837 to 1915, measured in the drafts from 1858 to 1936 (*n* = 53,503). Mean adult height reflects a crude indicator of net nutritional status, a proxy for currently known measures of stunting and wasting. The results reveal a cycle of stagnation and decline in average height at the age of 21 for those born from the 1850s to the 1870s and a subsequent positive secular trend to exceed baseline levels. The coefficient of variation shows how inequality in height followed an opposite pattern, with an increase in the mid-nineteenth century and a subsequent decline, with an overall decline. The great migratory wave towards Latin America (1880–1930) barely affected the area studied here. The available evidence on the occupations and educational level of the recruits reveals a ranking in average height related to family background and personal income, educational level and literacy, propinquity to food and ownership and/or management of land. Therefore, socioeconomic status largely predicted adult height in Spanish men during the period. Reducing absolute poverty and increasing access to education remain cornerstones to reducing malnutrition, even in the current world.

## 1. Introduction

The study of socioeconomic inequality has become a hot topic in the social sciences, and especially in economics, in recent years. In the last decade, interest in the subject has even transcended academic boundaries, drawing increasing attention in politics and in the media. 

Economic and social history is not alien to this phenomenon (See, among others, [1,2,3]). One of the most prolific research fields in recent decades, anthropometric history, that is, the study of anthropometric variables—especially height—as indicators of net nutritional status or biological standard of living in populations of the past, is not oblivious to the interest in the study of inequality. Since its inception more than four decades ago, a good part of the works belonging to this current research focused their attention on the analysis of differences in height, occasionally to other anthropometric variables, and their changes over time. Over decades, aspects such as the differences between social classes, ethnic groups, occupations, professional skills, literacy or educational levels were a regular part of the anthropometric research agenda [4,5,6,7]. Therefore, it can be said without exaggeration that the study of inequality is at the core of anthropometric history. 

In addition to the study of socioeconomic differences, in the last two decades, a growing literature is devoting monographic attention to the study of anthropometric inequality and its relationship with economic inequality, using the coefficient of variation (hereinafter CV) (or Pearson’s Coefficient of Variation or Relative Standard Deviation), the standard deviation divided by the mean, often expressed as a percentage, as the main indicator [8]. Although the standard deviation could be considered an intuitively useful indicator to measure anthropometric inequality, some biomedical research suggests that it increases with mean height [9]. 

Literature using the CV documented a wide degree of positive correlation between anthropometric and income inequality. In this sense, some studies [10,11,12,13,14,15,16,17] support the use, under certain conditions, of anthropometric inequality as a proxy for economic inequality, especially in the absence of solid economic data. However, as much of this literature acknowledges, the relationship is far from being automatic and depends on other economic and non-economic factors, such as income levels (and especially poverty levels), relative prices, nutritional demands from diseases and physical workload.

Spanish anthropometric literature participates in the aforementioned international historiographical trend, and, especially in recent years, some works have begun to devote monographic attention to biological and nutritional inequality. Thus, an increase in height inequality, differences among occupations and inequality within groups (such as farmers and artisans), related to growing income inequality, an increase in relative food prices and lower incomes in some socio-occupational groups, are documented in the cohorts born at the end of the 18th century in central Spain [18]. At the same time, a gradient in height related to the access to land property, educational levels, professional skills, access to animal proteins and family background is found for the same geographical and historical context [18,19]. 

In a case study on the Catalan town of Igualada [20], a middle town (10,000 to 15,000 inhabitants in the period studied) with an important process of industrialization in the 18th and 19th centuries, a drop in inequality measured with the CV was detected among conscripts—both among those born in that town and among those with other origins—born in the 1840s, a subsequent increase among generations born between approximately 1850 and the mid-1870s and a subsequent decline and stagnation until those born in the second decade of the 20th century. General patterns were not exempt from strong fluctuations, especially in the central decades of the 19th century.

In a case study for the Rio Tinto mining area (Huelva, Andalusia) [21], evidence of inequality increase (measured with the CV) during the central decades of the 19th century was found. A subsequent drop was also clear, followed by a temporal increase in the last years of the 19th century and the first years of the 20th century, and a final fall and stabilization until the cohorts born in 1915. 

In the case of the Valencia region [22], a gradient from non-manual skilled workers at the top to manual and day laborers at the bottom, passing through non-manual semi-skilled and farmers as intermediate categories, was detected for the cohorts born from 1859 to 1967. At the same time, the evolution of the CV shows a clear decline in the last decades of the 19th century, a subsequent slower decline in the early 20th century, a rebound in cohorts born around the Civil War and a slight decline thereafter. 

For Extremadura [23,24], both the Gini index and the CV shows an increment for the cohorts born in the decades of 1850 to 1870, a subsequent fall in the CV until the last decade of the 19th century, following stagnation later and finally a slight drop in the cohorts born from the mid-1920s onwards.

In a recent study [25], an extensive database from Mediterranean Spain (basically the regions of Valencia and Murcia) detected large and growing nutritional inequalities in the mid-19th century, a sharp decline in the decades straddling the late nineteenth and early twentieth centuries and a slight subsequent increase that began with the cohorts that suffered the Civil War and the postwar periods. At the same time, a clear and persistent gradient related to educational level and literacy was detected. 

A case study for the municipality of San Bartolome in Lanzarote (Canary Islands) confirms the trend found in other peninsular regions for the period comprised between the 1880s and 1910s (birth cohorts) [26].

For Spain as a whole [10], a net increase in CV by socio-professional groups was documented for those generations born during the first half of the 20th century. Specifically, it can be considered as a result of what happened in the early years of the century, during the First World War and during the Civil War (1936–1939) and the post-war period (1940s).

This article is part of a current research that focuses on the evolution of inequality in male height in relation to socioeconomic inequality and its potential determinants. In particular, the research question is to evaluate the impact of economic inequality on anthropometric (height) inequality as an indicator of the net nutritional status or biological wellbeing of the population.

It focuses on a large area of central Spain, including hand-collected data (*n* = 53,503) on conscripts born between 1837 and 1915 and drafted between 1858 and 1936 (both included) in 28 localities from the provinces of Madrid, Toledo, Ciudad Real and Guadalajara, currently Community of Madrid in the first case and Castile-La Mancha in the others.

The paper is structured as follows. After this introduction, Section 2 is devoted to presenting the main characteristics of anthropometric data, its sources and the methodology used in the calculation of the series. In Section 3, the main results are presented and discussed. Section 4 deals with interpreting the findings in the light of the results offered by economic history and historical demography for the geographical and historical context. Finally, in Section 5, I provide the main conclusions.

## 2. Materials and Methods

In the period on which this work is focused, the base of the Spanish army troop was made up of young men from the annual call-ups, in which all males of a certain age were mandatorily included, physically examined and measured. Therefore, this study also includes those subsequently exempt due to physical or social reasons, short stature or those who, at a later stage of the process, paid a substitute or an amount of monet to avoid the “blood tax”. In the call-ups between 1858 and 1936, only in the years 1900 and 1906 was there no draft due to the change in the draft age. In 1875 there was not either, but the data from the October 1875 call-up are used since they correspond to the generation born in 1856. Other special cases that deserve to be mentioned are the existence of two call-ups in 1885, due to a change in the age of conscription, and the fact that in 1873 and 1874, conscripts were not measured. 

The first phase of the conscription process was carried out at the local level and was divided into several stages. Therefore, the replacement operations were developed in the local councils, which is why, if the documentation is preserved, it is usually kept in the municipal archives. In the sample of communities included in this work, the exception to this rule are the data from Bustarviejo, Camarma de Esteruelas, Loeches, Paracuellos del Jarama, Torrelaguna, Villaconejos, Villamanta and Villamantilla, conserved in the regional archive of the Community of Madrid, and those from Almorox and Aldeaencabo de Escalona, preserved in the Toledo Provincial Council (Diputación Provincial) archive. First, a list (*Alistamiento*) which included everymen at a specific age (in the period considered here, depending on the time 19, 20 or 21 years) resident in the municipality, based on the information from the parish books, the civil registry and other municipal registers was drawn up. Then, in a second step, the order in which the males would be examined was assigned by draw (*Sorteo*). Finally, in that order, they were called to be publicly measured (barefoot) and examined by doctors (this step of the process was called *Clasificación y Declaración de Soldados y Suplentes*) and the allegations they wanted to present heard. After this procedure, they were declared soldiers, totally exempted or temporarily exempted but subject to examination in the next year’s call-ups. Quite frequently, as we will see, their occupations and literacy levels were also recorded. This recruitment process was maintained, without changes in the fundamental aspects, in the period studied here. 

**Figure 1:**
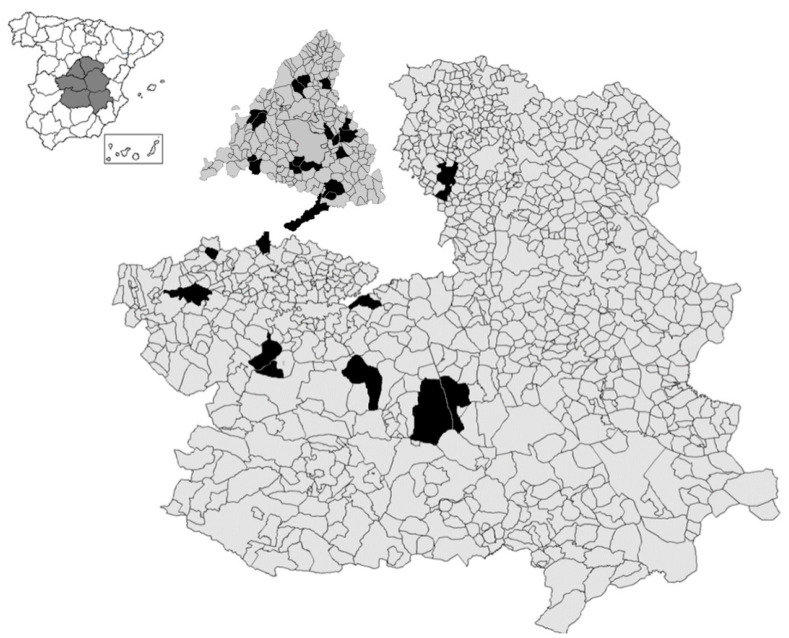
Communities included in the database and location in central Spain.

This work is based on the source, the *Expedientes Generales de Reemplazo*, hereinafter *Expedientes*, in particular on the information included in the *Clasificación y Declaración de Soldados y Suplentes.* The vast majority of Spanish anthropometric history is based on this local source, especially for periods prior to the second half of the 20th century. Specifically, for the sample of localities located in the Madrid region, an essential part of the total studied in this paper, see reference [27]. The data comes from the *Expedientes* made between 1858 and 1936, corresponding to the generations born between 1837 and 1915, in 28 communities located in central Spain (see Figure 1). 

The criteria followed in the selection of localities for the sample were the following: the existence of continuous (no gaps) or almost continuous series (the main gaps are found in the series of Alcazar de San Juan (1858–1896, 1901–1911, 1928–1936), Aldeaencabo de Escalona (1858–1881 and 1924–1936), Almorox (1858–1864 and 1921–1936), Getafe (1889–1896 and 1905–1918), Leganés (1858–1882), Fuenlabrada (1864–1879), Torrejón de Ardoz (1858–1896 and 1904–1912) and Torrelaguna (1864–1872)) and the representativeness of the different geographical, demographic and economic settlements existing in central Spain. The sample of localities includes from small rural villages, with just a few of hundred inhabitants, to provincial capitals (case of Guadalajara), as well as rural communities with different population sizes, including agro-towns (case of Campo de Criptana) and urban municipalities (usually with more than 10,000 inhabitants) specialized in a mix of agrarian and tertiary (Alcazar de San Juan, Alcalá de Henares or Aranjuez) or agrarian and industrial activities (Talavera de la Reina) and acting as regional capitals. 

Spanish *Expedientes* are one of the best existing military sources at the international level for the study of anthropometric history. First, they proceed from conscription (universal replacements) and, therefore, there are no biases related to the conscripts’ social origin. Second, the usual problem in military sources of the existence of truncation or censoring below the minimum height requirement, in the left side of the distribution, does not exist since all heights (including those below the minimum established to be considered fit) were recorded. Third, the source often includes quality information on a number of physical and socioeconomic traits of the conscripts. 

However, despite its advantages, this source could also present some problems and shortcomings that limit its usefulness or skew the information. First, changes in the age of conscription (the changes took place in 1885 (second call-up) from 20 to 19 years in 1885, from 19 to 20 in 1901 and from 20 to 21 in 1907) and, therefore, measurement could cause a lack of homogeneity in the series. Second, high numbers of draft dodgers may also condition the results if, as is foreseeable, most of them overcame the minimum height required, and other personal features, to be considered fit. Finally, it is worth checking whether the data comply, even approximately, the usual assumption of normality attributed to the distribution of adult human height. 

Regarding changes in the age of conscription, the problem was recognized and corrected in the Spanish anthropometric literature. In recent years, all the papers based on these sources dealt with this problem in different ways. In the main regional series, in a seminal article dealing with the problem of height for age standardization, Martínez-Carrión and Moreno-Lázaro [28] (p. 152, Table 5) found growth of 1.1 cm between 19 and 20, 0.5 cm between 20 and 21 and, therefore, 1.6 between 19 and 21 for Southeastern Spain and Castile and Leon. García-Montero [27], following Martínez-Carrión and Moreno-Lázaro’s methodology for an area partially (rural Madrid) coincident with that of this paper, obtained, depending on the period, growth of between 2.51 and 4.84 mm between 20 and 21 years, 7.94 mm between 19 and 20 years and 12.78 mm between 19 and 21 years. Ramón-Muñoz [29], following an approach based on the 50th percentile, established a pattern of growth of 1.25 cm between 19 and 20 years, 0.7 cm between 20 and 21 and 1.95 cm between 19 and 21 years in rural Catalonia. With the same methodology, Cámara et al. [25] calculated a growth of 1 cm between 19 and 20 years and 0.4 between 20 and 21 years, with a total of 1.4 cm to standardize the series to 21 years for Mediterranean Spain (basically Murcia and Valencia regions). To standardize the series to 21 years, following Martínez-Carrión and Moreno-Lázaro [28] (p. 152, Table 5), I will use the data from Table 1, which includes the average annual growth of those conscripts found temporarily unfit. That is to say, those with a height between 1500 mm and a variable figure depending on the range between 1535 and 1570 mm, that were required to be measured annually within 3 years of their call-up to check if they had reached the minimum height. With this table, I calculate the growth, depending on the age draft requirement in the different periods, between 19 and 20 and 20 and 21 years. The results are similar to those obtained, with the same or different method, in the mentioned Spanish series for the same period.

With respect to the draft dodgers, the percentage of them was usually low, below 5%. Only exceptionally in 1897, in the midst of the Cuban War of Independence and on the eve of the Spanish–American War, and in the 1920s, during the colonial conflicts in Morocco, was there an increase in those figures. The figures reached almost 20% in the first case and between 5 and 10% in the second. However, it should be borne in mind, that some of the draft-dodgers were actually migrants who quite frequently, eventually, sent the corresponding draft certificate from their new place of residence. 

**Figure A1:**
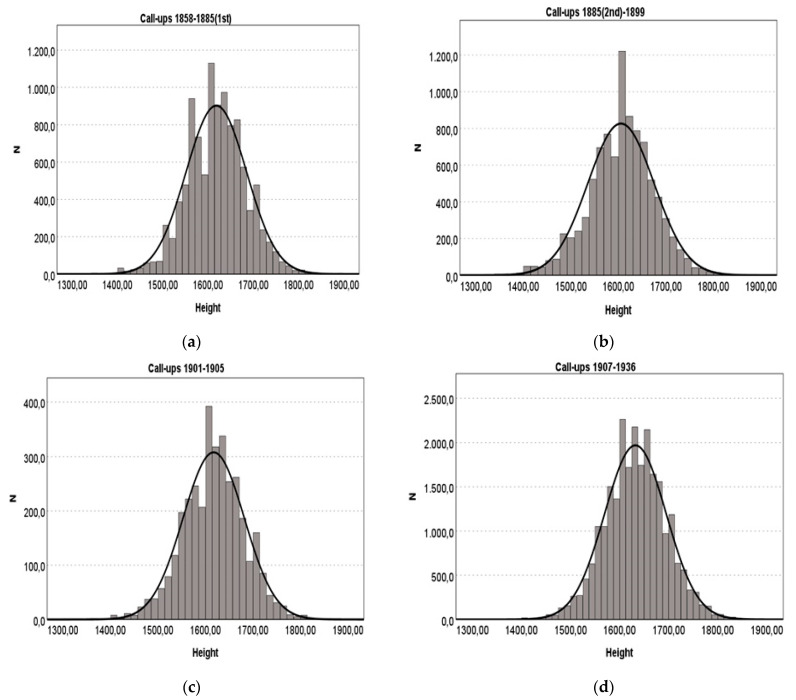
Histograms with the distributions of heights. Call-ups 1858–1936 (Source: built with data from the corresponding *Expedientes*). Data expressed in thousands with “.” and decimals with “,” in the axes.

Finally, a look at the histograms (see Figure A1 in Appendix A), corresponding to the periods in which the appeal was made at the same age, shows how height remained approximately as a Normal or Gaussian statistical distribution, with the characteristic bell shape. Only some heaping and rounding, frequently quite symmetric and therefore with little or no impact on the results [30] (p. 161), was detected around some figures such as 1550, 1600 or 1650. 

## 3. Results 

**Figure 2:**
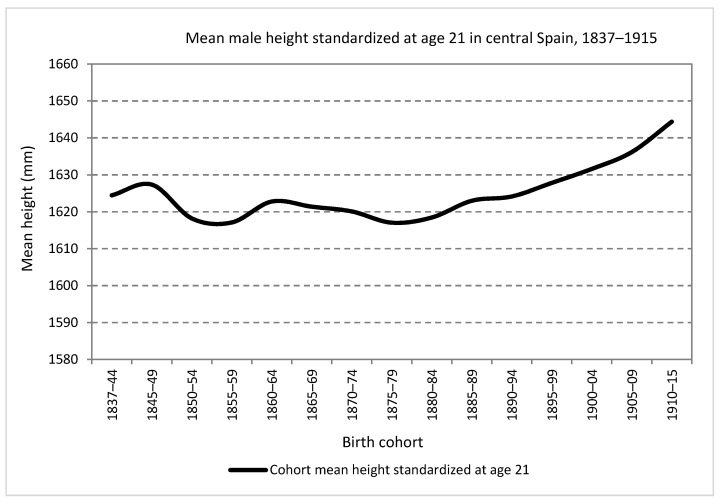
Mean male height at age 21 in central Spain, birth cohorts 1837–1915 (Source: calculated with data from the *Expedientes*).

Figure 2 show the evolution of mean height, the standardized series at 21 years, for men born between 1837 and 1915 in central Spain. As can be seen, those born in the central decades of the 19th century suffered various phases of decline and stagnation in their physical stature. Consequently, the starting levels of the series, around 162.5 cm, were not definitively exceeded until the generations born in the early years of the 20th century. Specifically, cycles of decline were detected between those born between 1845 and 1860 and between 1865 and 1880. In the second half of the 1870s, there was an inflection point that marked the beginning of the secular trend of growing average height in Spain. This trend continued, with the exception of the cohorts growing during the Civil War and the postwar decade [22,23,31,32,33,34,35], until the generations born at the end of the 20th century.

These results, in their general outlines, were also documented for other Spanish regional series [23,27,29,31,33,36,37]. As a characteristic of the central Spain series, it is worth mentioning that the values are in the lower or middle range of the Spanish anthropometric ranking together with those of the regions of Extremadura, Castile and Leon and Murcia and below those for the Canary Islands, Catalonia, Biscay and the Valencia region. In addition, it shows a greater deterioration in the generations born in the central decades of the 19th century with respect to other regions, with the only exception of Castile and Leon. 

The explanation of the general trends, stagnation and decline during the central decades of the 19th century was attributed to the costs associated with the beginning of economic modernization in the form of economic inequality and poverty, frequent subsistence crises, a worsening of health conditions at early ages as shown by the increase in childhood mortality rates and a slow reduction in child labor motivated by economic and social factors in addition to a low and late allocation of public resources for primary education. Most of these factors, acting in the opposite direction, would explain the beginning of a positive secular trend from the cohorts born in the last decades of the 19th century. 

**Figure 3:**
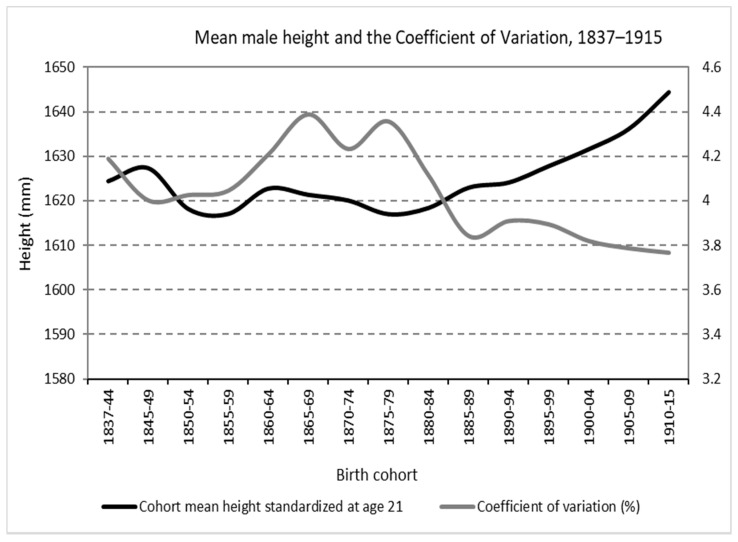
Mean male height at age 21 and coefficient of variation in central Spain, birth cohorts 1837–1915 (Source: calculated with data from the *Expedientes*).

**Figure 4:**
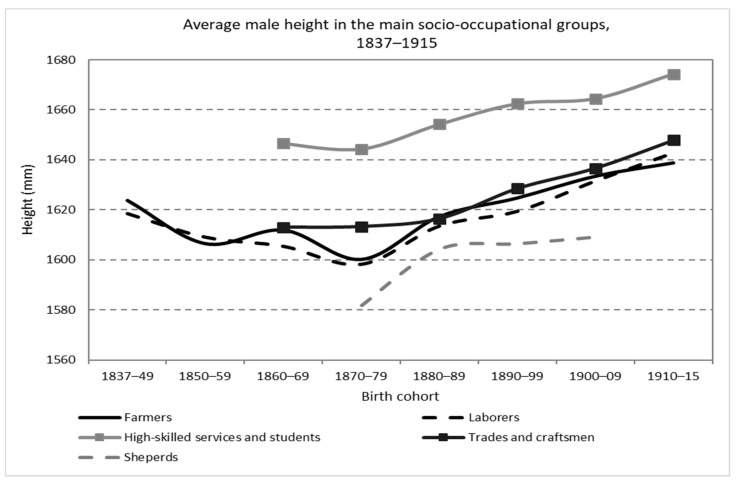
Average male height in the main socio-occupational groups, birth cohorts 1837–1915 (Source: calculated with data from the *Expedientes*).

In Figure 3, it can be seen how the mean male height largely followed an inverse pattern to that of the coefficient of variation. While the central decades of the XIX century show stagnation and fall in physical stature, at the same time, a growth of the CV is observed. Both forces, the drop in height and, above all, the increase in the standard deviation, push in the direction of greater anthropometric inequality. On the contrary, for the generations born in the final decades of the 19th century and the beginning of the 20th, the continuous increase in the mean height and the fall of the standard deviation first and its later stagnation resulted in a decline in the CV. Indeed, an increasing mean height coexists with a fall, accelerated first and slower subsequently, of the CV until it reaches levels lower than those of the beginning of the series. These results suggest exploring a starting hypothesis, the possible relationship—basically inverse—between the trend in economic inequality as a determinant, at least partially, of the evolution of average adult height. A relationship between an input—the distribution of economic resources across the society and, therefore, nutrients, health environment, access to medical care and child labor—and an output that is the result of the effect of the combination of said outputs on the human body size was determined. Beyond a general perspective on the evolution of height inequality, the richness of information included in the *Expedientes* makes it possible to address biological–nutritional inequality from other specific angles. The *Expedientes* also quite frequently included information about the occupation of the conscripts. Ideally, the father’s occupation would be more relevant as a determinant of the recruits’ nutritional status. However, as in most of the anthropometric literature based in military sources, we can assume that social mobility was still low (especially in rural areas) and the conscript’s profession could be relevant in terms of nutrition and health and, therefore, physical growth, from adolescence. Specifically, in 28.59% of cases (*n* = 13,801) the occupation was recorded. On the basis of the 130 different professions coded, Figure 4 show the evolution of mean height in the main (most numerous) socio-professional categories. 

It should be noted that all series, based on hundreds of observations for each decade (the only exception is the case of shepherds with a few dozens per decade, but always more than 30 observations per decade), share a trend of stagnation or decline in the middle decades of the 19th century, even for highly skilled service workers and students, and a subsequent positive secular trend and recovery. Second, a clear and persistent gradient was detected between a group with taller heights, highly skilled workers, this category includes professions related to the health sector (doctors, pharmacists, veterinarians and practitioners), the clergy, teachers, and white-collar and highly qualified workers in general (among others lawyers, surveyors, accountants or journalists) and students, another group with lower heights, around 3 cm shorter, but quite similar between them, composed of laborers, farmers and trades and artisan workers, and finally, a group with the shortest physical stature, between 1 and 3 cm shorter than the previous category, represented by the shepherds. 

To explain the high nutritional levels detected among highly skilled workers and students, it is worth referring again to the explanation already presented for the case of students: their family background and the possibility of obtaining a higher income during their adolescent spurt and final years of growth until the end of physical maturity. 

Regarding the group of professions with an “intermediate” height, a small but almost always positive gap was detected between farmers and day laborers. This gap is not strange if we consider that the condition of farmers, despite being very diverse, implies access to ownership and/or management of land while the laborer is simply someone with little or no access to land and capital goods, e.g., a labor offeror with a casual employment relationship. In any case, the small dimension of the gap is more striking, in many cases less than 1 cm. However, this narrow margin is quite coherent with previous results found in some Spanish regional series and may be related to the fact that the vast majority of farms were small, tiny or farmers were not even owners at all; most of them were also frequently subject to growing land rents and impoverishment, e.g., during the Long Depression (*Depresión Finisecular*).

For its part, the trades and craftsmen series is located in the middle zone of the ranking throughout the period with information. However, given the heterogeneity of the professions included in this category (89 different professions), it is worth disaggregating in more detail and analyzing the differences between some of the professions with more representation in the database. The level of income, qualification, access to food (especially animal proteins), the necessary physical strength and even self-selection based on other physical characteristics are factors that the literature has resorted to when explaining the differences in average height between trades (See, among others, references [18,19,31,38,39,40,41,42,43]). 

**Figure 5:**
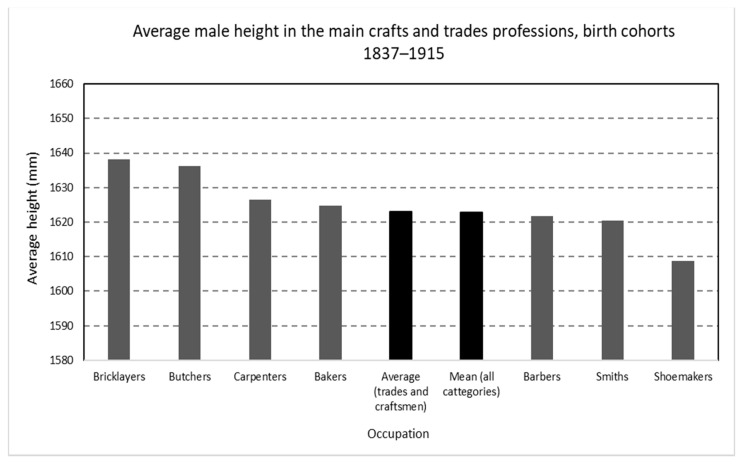
Average male height in the main crafts and trades professions, birth cohorts 1837–1915 (Source: calculated with data from the *Expedientes*).

In Figure 5, we can see the gradient of the main categories. The general average height for trades and craftsmen is slightly above the mean height of the whole database, just as in the case of generations born at the end of the 18th century in the same area [18,19]. Both bricklayers and carpenters are above the average; therefore, they seem to corroborate the hypothesis of a positive relationship between physical strength needs and taller heights. Stranger, is the fact that blacksmiths appear, albeit narrowly, below average. In the same way, bakers and especially butchers support the usual positive link between propinquity to food and, in particular, animal proteins and stature above the average. Among the categories with a lower than average height, in addition to blacksmiths, there are barbers and shoemakers. Occupations that do not require special physical abilities could even be related to self-selection based on the absence of physical traits such as strength or tall height. These occupations do not allow direct access to food and are not related to skills or income, in principle, higher than those of other trades and artisans. 

In sum, the ranking found in this period is not very different from the one found in the same area for the generations born in the last decades of the 18th century [18,19]. Just the relative position of blacksmiths can be considered somewhat odd. 

**Figure 6:**
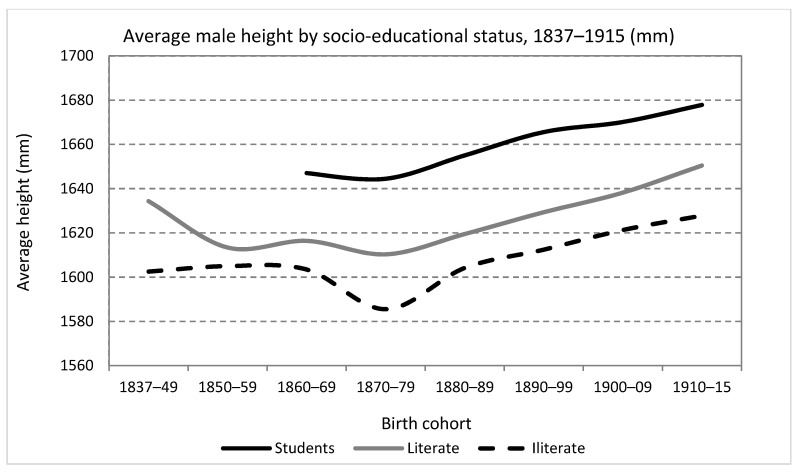
Average male height by socio-educational status, birth cohorts 1837–1915 (Source: calculated with data from the *Expedientes*).

With regard to literacy, in 39.74% of the cases (*n* = 19,181) it was recorded whether the conscripts could read and write or not. Furthermore, as will be seen immediately, in a significant number of cases, the occupation of the conscript was also recorded, including those whose “profession” was that of a student. Figure 6 show how the educational level marked a persistent and relatively stable gap in average height. The “students” had a higher net nutritional status, between 2.5 and 3.5 cm, compared to those who were simply literate (excluding students). Finally, the series with the average height of the illiterate always appears, with levels between 1 and 2.5 cm shorter than the literate conscripts. These results are similar to those found by Cámara et al. [25] for Mediterranean Spain in the same period. 

Another important characteristic of the figure is that, despite the persistent differences, the different series seem to show similar patterns in the long-run stagnation or decline in the central decades of the 19th century (although to a lesser degree in the series “students”) and recovery and a positive secular trend since those born in the 1870s. This seems to suggest that the factors, or at least some of them, that explain the “nutritional crisis of the mid-nineteenth” century also had some effect on the upper classes. The most obvious candidate is those factors related to children’s health with an exogenous (not socioeconomic) origin. 

The explanation of the differences found in Figure 6 has attracted constant attention in the anthropometric literature. In societies with a low educational level, with a non-compulsory (or non-respected compulsory) basic education and with a wide spread of child labor, the simple fact of being literate is already indicative of the higher economic and educational level of parents. Furthermore, to the extent that in Spain, the percentage of non-literate men has been progressively decreasing since the second half of the 19th century, it is probable that those who are not literate came more and more from the poorest strata of society. However, there is no growth in the gap except in the 1840s and during the general drop during the 1870s, and the secular trend shows a similar profile than in the rest of the series. 

Besides, in a society in which basic education was still a relatively scarce good, the fact of knowing how to read and write necessarily had to imply in many cases, especially in the urban world, greater possibilities of access to more skilled and, therefore, better-paid professions. It could also be a key tool in the case of emigrants to access higher income in the destination, often urban. That is, regardless of social origin, physical growth in adolescence and early youth could be favored by higher income and less physical effort in those young men with a higher educational level. 

When it comes to students, their privileged status should come as no surprise. A person classified as a student between the ages of 19 and 21 shows access to secondary or higher education, which, in the context of a society with a high number of illiterates, means belonging to a privileged minority with access to such studies. In addition, it should not be overlooked that, in some cases, the “student” label could simply reflect the socioeconomic condition of the son of a well-to-do family, not necessarily linked to the achievement of formal studies. 

## 4. Discussion

This section is devoted to trying to answer two interrelated questions: what factors explain the results obtained in Section 3? What relationship could exist between the trajectories of height inequality and socioeconomic inequality? 

As mentioned in the introduction, a large literature demonstrated the sensitivity of the distribution of height to the distribution of income. However, causality can also work in the opposite direction. In other words, under certain conditions, height inequality can also be a predictor of income inequality. 

Changes in income distribution can affect height distribution in several ways. In a society in which the vast majority of its members have not reached their maximum genetic growth potential—as is the case of the one studied here—a redistribution of income favorable to the poorest groups in the absence of changes in other factors should mean an increase in their heights due to an improvement in their nutrition. At the same time, the high-income groups, closer to their genetic potential, will be little or not, in any case, much less, affected [4,44]. This reasoning also works logically the other way around, if the redistribution of income is favorable to the richest, it will mean little or no gains in the average height of that group as well as a greater deterioration in the net nutritional status of the poorest. Economic theory argues that the income elasticity of demand for food is less than 1 and, therefore, in the face of a certain increase in income, the increase in spending on food will always be lower and decreasing (Engel’s Law). In addition, this economic law is more strongly fulfilled in staples such as cereals, tubers or legumes. 

Changes in the relative prices of food can also affect social differences in height since the sensitivity of consumption to variations in the prices of food (in economic terms, the “price elasticity of demand”) is higher among social groups with low incomes [10] (p. 111). This reasoning also works in the opposite direction. 

However, as it is widely known, the determinants of average height as an indicator of net nutritional status include other factors beyond nutrient intake. In particular, the key importance of nutrients demanded from diseases and physical labor is emphasized. Obviously, these factors can be partially influenced by income (and, therefore, by economic inequality) and, at the same time, also affect the relationship between economic inequality and the biological–nutritional inequality shown by height. 

In the case of central Spain, how did the relationship between economic and anthropometric inequality evolve in the period between the mid-19th century and the outbreak of the Civil War? To what extent was anthropometric inequality conditioned by the trajectory of economic inequality?

To answer these questions, the absence of local or regional information, for example, in the case of indicators such as the Gini index, Williamson index or absolute poverty, is a major obstacle. The validity of the use of information at a national level for a regional case should not be taken for granted. Therefore, the reasoning that follows must be taken only as a very tentative reflection. Future research, based, for instance, on regional data on economic inequality, could change or refine this part of the work.

The start of modern economic growth in its Kuznetsian definition—sustained and linked to structural change—is usually located in the Spanish case around 1850 [45,46]. However, this process coexisted with an increase in economic inequality in the period studied here [47]. Whether we look at an indicator such as the Williamson index (the relationship between the GDP per worker and the unskilled wage), the wage Gini (dispersion of wages) or the Gini index, the net balance points to an increase in economic inequality. We took the year of birth of the last cohort of conscripts (1915) or the year in which it was measured (1936) as a reference for the end of the economic inequality series. A more in-depth look at the series shows the existence of different dynamics according to the subperiod. For instance, if we observe the Williamson index after a slight decline in the early years, there was an increasing trend between 1865 and World War I, with particularly clear periods of growth between 1865 and 1885, 1895 and 1905 and during World War I. Subsequently, between the end of World War I and the beginning of the Civil War in 1936, a clear drop in inequality was observed. A very similar profile, with minor departures, was found in the Gini index. On the other hand, if we look at the internal dispersion of wages (wage Gini), the main difference is related to a continuous escalation of wage inequality that reaches up to the Civil War [47] (p. 294). 

Therefore, the resounding decrease in anthropometric inequality during the period, regardless of whether we take as a reference the birth year or the date of conscription, is clearly at odds with the net balance in terms of economic inequality. In detail, it seems clear that the strong increase in anthropometric inequality found in the cohorts born roughly between 1860 and 1880 (and growing between roughly 1880 and 1900) is consistent with an increase in economic inequality, especially during the first years of life. However, in the period between 1895 and World War I (always referred to as birth cohorts), while economic inequality grew, height inequality, except in the early 1890s, had a slight decline. In the same way, in the period after World War I, while economic inequality decreased rapidly, the CV was stagnant for the conscripts, still growing until the outbreak of the Civil War (1936). 

**Figure 7:**
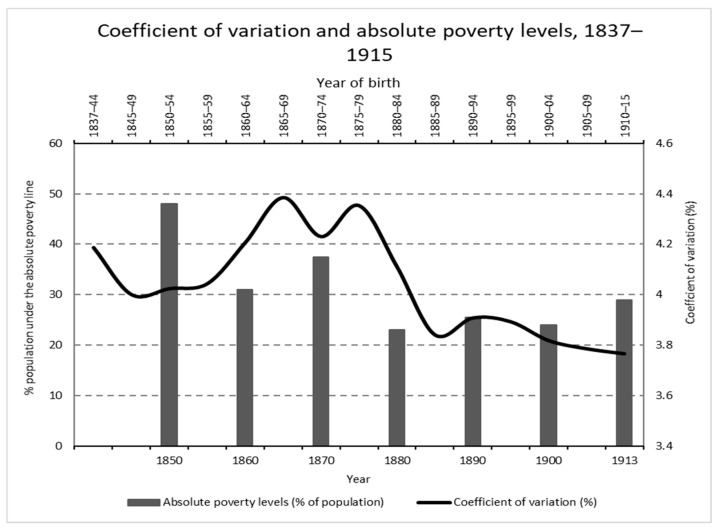
Coefficient of variation in height in central Spain and absolute poverty levels in Spain, 1837–1915 (Source: calculated with data from the *Expedientes* and [47]). No data for absolute poverty levels were available before 1850.

The picture is more complex if we insert absolute poverty dynamics [47] (p. 309) (absolute poverty is defined as $2 (1985 Geary–Khamis dollars) per day per person). In fact, the potential negative effects on height inequality of an increase in income inequality can be neutralized if, at the same time, there is a general increase in income or, at least, an increase in the income of the poorest strata of society. According to Prados de la Escosura [47], absolute poverty underwent a great fall between 1850 and 1935, from almost 50% to 12% of the total population. As can be seen in Figure 7, the first important decline took place from 1850 to 1880, with an upturn in the 1860s, few changes afterwards until a temporary increase during World War I and finally, going beyond the data shown in Figure 7, a sharp fall in the last two decades until the Civil War, in particular during the years of the Second Spanish Republic (1931–1936). Therefore, absolute poverty could be an important reliever of the impact of economic inequality on height inequality in the whole period, but undoubtedly, it was not enough to counteract it in some sub-periods.

Regarding relative prices, as a result of the industrialization process, there was a general trend of decrease in the price of industrial goods with respect to food [48] (pp. 58, 95–101). The general trend was particularly intense during the frequent subsistence crisis that happened between the 1840s and the 1870s. The subsistence crisis hit inland Spain with particular intensity, including the territories studied in this paper. Thus, that force seems to have always pushed in the long run toward greater anthropometric inequality. However, two important exceptions have to be considered. First, during the Long Depression (roughly 1873–1896), in particular with its aftermath of deflation in the agrarian products in the 1880s. In that period, small agrarian producers were hit by low agrarian prices and suffered serious impoverishment that boosted migrations. In other words, against the usual rule, an important part of the left side of the income distribution, composed of small agrarian producers, were harmed by a decrease in the relative price of food. However, it is important to point out that the great migration wave from 1880 to 1930 was, in the Spanish case, above all, a regional phenomenon, and the origin of the Spanish emigrants was far to be homogeneous in geographical terms. The communities studied in this work belong to provinces (included in the present regions of Castile-La Mancha and Madrid) that were systematically among those suffering fewer migration rates during the period. They contributed an insignificant number of emigrants (usually less than 1% of the total migration in each case and with a rate of migration less than 1 per 1000) to the flow towards the Americas. According to Sánchez-Albornoz [49] (p. 21), Yáñez-Gallardo [50] (pp. 233–241) and Sánchez-Alonso [51] (pp. 205–231), the main regions of origin of the emigrants were, by far, located in northern Spain (especially Galicia and Asturias) and the Canary Islands. Other regions such as the Basque Country, Catalonia or the provinces of Leon (provinces close to the border with Portugal) also had significant migration flows to the Americas. Therefore, I assume that this phenomenon had a very limited impact (if any) on our series. Additionally, relative food prices cheapened in the period 1914–1936, when some of the conscripts from the last generations were still growing. 

In summary, there are periods in which all the economic factors point in the direction of a reduction in anthropometric inequality, as in the case of the 1850s—except for relative prices—or the period 1918–1936; periods in which they coincide in pushing in the direction of greater height inequality, as in the case of the 1860s or during the First World War; and finally, periods, as in the case of the years between 1870 and 1914, in which the net balance between different forces, acting in different directions, is not clear. 

**Figure 8:**
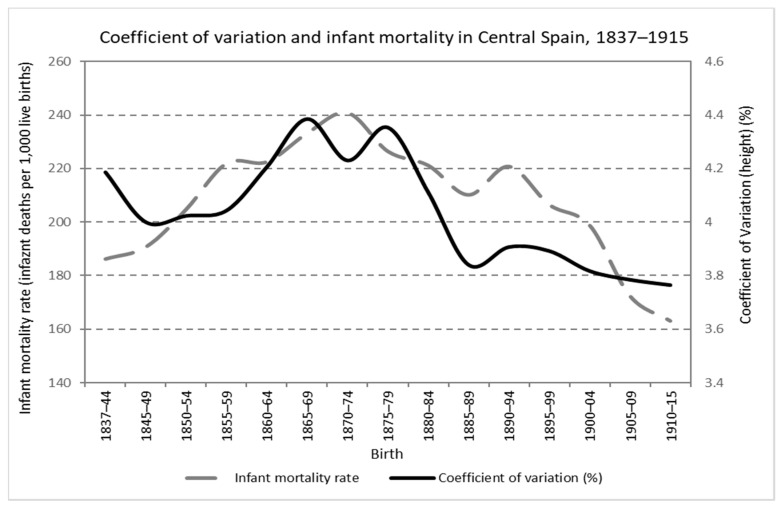
Coefficient of variation and infant mortality in central Spain, birth cohorts 1837–1915 (Source: calculated with data from the *Expedientes* and [52]).

Last but not least, as already mentioned, causes that are different from the strictly economic ones do have an impact on physical growth. They are two fundamental factors: nutritional demands from diseases and physical workload. In the period studied in this work, it is clear that during the central decades of the 19th century, there was a worsening of health conditions in infancy and childhood, as evidenced by the increase in mortality rates at an early age [52]. In particular, unlike the other variables mentioned, as can be seen in Figure 8, regional information focused in central Spain exists in this case [53,54], and the series is categorical to show the deterioration, and the later fall, of early age mortality rates between 1850–1880.

Subsequently, from the last decades of that century, a secular trend towards a fall in mortality began, which shows a constant improvement in living conditions in infancy and childhood. To what extent this drop was related to an improvement in economic conditions, other non-economic factors or a mixture of both has been the subject of the classical debate on the causes of the secular decline in mortality rates during the demographic transition. For this research, it is enough to underline that, overall, the profile of the evolution of mortality at an early age coincides with that of inequality in height. In other words, the height inequality increase (and later decrease) could also be related to the different social impacts of some diseases at early ages. Indeed, upper classes lived with better environmental conditions and hygiene and enjoyed easier access to medicines and medical care at a time when the medical revolution derived from the discoveries of Koch and Pasteur began to increase the importance of access to medicine as a determinant of health and longevity. At the same time, from the late 19th century onwards, the growth of family incomes, the drop in fertility and its consequences for the allocation of intra-family resources and the improvements in public sanitation reduced social differences in access to medical care and environmental health conditions. 

It is more difficult to speculate on the evolution of the physical workload demands and their impact on the height of the conscripts. In the absence of hard evidence, I can just argue that child labor had to be negatively related to the general economic performance, in particular family incomes for the poorest strata of society, and the extension of primary education. In this sense, the general decrease in absolute poverty (even with some upturns before 1914), and the slow but progressive diffusion of primary education, as shown by literacy rates, point to a vague decrease in child labor since the last decades of the 19th century. Therefore, this factor would also have driven a fall in social differences in height in the period as a whole and, especially, since the end of the 19th century. 

## 5. Conclusions

The relationship between height inequality within a society, or a part or group of it, and economic inequality, has attracted increasing attention in anthropometric history in recent years. The general evidence points to a high correlation in the trends of both variables, with causality ranging from income inequality to the social distribution of height. However, this relationship is not always universal, and the connection is complex due to the possible effect of other variables. This work is a good example of it.

In the case of this research, focused on central Spain, the trajectory of the coefficient of variation indicates a decrease in social inequality in height in the long run among the generations born between 1837 and 1915. However, beyond the general balance of the entire period, there is evidence of a cycle of a strong increase in inequality in the central decades of the nineteenth century and, specifically, among the generations born in the decades from 1850 to 1870. For its part, the analysis of the differences between professional categories detects a persistent and fairly constant gradient in its magnitude among the main socio-professional groups. This gradient is related to variables such as family economic status, own personal income, access to property and/or land management in the case of the primary sector, educational level and literacy and direct access to nutrients in the occupation. At the same time, among those young men who were employed in manual trades and craftsmen, the possible selection in the access to certain professions in which a need for greater bulk was foreseeable, as in the case of bricklayers and carpenters, is also observed as a potential explanatory factor for the differences. 

The results reinforce the findings for the same area at the end of the 18th century, those obtained in other Spanish regions for the 19th and 20th centuries and, in general, those found in the international literature. 

Therefore, although economic inequality, which is shown to be potentially explanatory in some periods in the series, is a factor to consider, the relationship is not automatic or direct and, in other phases, there is a certain divergence that could be explained by another key economic or non-economic factors, such as poverty and income levels, morbidity impact and physical workload. However, further research in the future, as far as possible based on regional or local indicators of economic inequality and using statistical or econometric models, should test and enrich, qualify or refute the tentative reflections included in the discussion section about the dynamic relationship between economic inequality and anthropometric inequality. 

## Figures and Tables

**Table 1:** Increments in average height by age of conscription (in mm). In 1885, due to the change in the age of conscription (from 20 to 19 years), there were two call-ups.

	1858–1875	1876–1885 (1st)	1885 (2nd)–1899	1901–1905
19 to 20 years	---	---	8.71	---
20 to 21 years	3.15	5.02	5.03	3.82
19 to 21 years (sum)	---	---	13.74	---
N	96	142	327	51

Source: review of temporarily exceptions included in the Expedientes of Alcalá de Henares, Almorox, Bustarviejo, Campo de Criptana, Fuenlabrada, Leganés, Loeches, Menasalbas and Miraflores de la Sierra.
